# Supplementary material for: Mechanisms governing the pioneering and redistribution capabilities of the non-classical pioneer PU.1
Source: Nat Commun. 2020 Jan 21;11:402. doi: 10.1038/s41467-019-13960-2 (PMC6972792; doi:10.1038/s41467-019-13960-2)
Supplement: Supplementary file 7 — Source data [file 41467_2019_13960_MOESM7_ESM.zip › Source_Data/FigureS5/FigureS5A_MotifsCommonPeaks/homerResults/motif24.similar.html]

motif24

## Information for motif24

A
C
G
T
T
C
G
A
A
G
T
C
G
A
T
C
G
T
C
A
A
T
G
C
G
C
T
A
G
C
A
T
G
A
T
C
G
C
T
A
T
C
G
A
C
G
T
A
  
Reverse Opposite:  

G
C
A
T
A
G
C
T
C
G
A
T
C
T
A
G
C
G
T
A
C
G
A
T
A
T
C
G
A
C
G
T
C
T
A
G
C
T
A
G
A
G
C
T
T
G
C
A
  

|  |  |
| --- | --- |
| p-value: | 1e-63 |
| log p-value: | -1.457e+02 |
| Information Content per bp: | 1.715 |
| Number of Target Sequences with motif | 235.0 |
| Percentage of Target Sequences with motif | 2.64% |
| Number of Background Sequences with motif | 186.3 |
| Percentage of Background Sequences with motif | 0.49% |
| Average Position of motif in Targets | 106.1 +/- 46.3bp |
| Average Position of motif in Background | 100.1 +/- 62.6bp |
| Strand Bias (log2 ratio + to - strand density) | -0.3 |
| Multiplicity (# of sites on avg that occur together) | 1.01 |
| Motif File: | file (matrix) reverse opposite |

### Similar de novo motifs found

|  |  |  |  |  |  |  |  |
| --- | --- | --- | --- | --- | --- | --- | --- |
| Rank | Match Score | Redundant Motif | P-value | log P-value | % of Targets | % of Background | Motif file |
| 1 | 0.885 | A T G C A T G C G T C A A G T C G C A T G C A T A G T C C G T A T G C A C T G A T A C G | 1e-60 | -138.236893 | 4.45% | 1.44% | motif file (matrix) |
| 2 | 0.771 | A G T C G C A T C G A T C G A T A T C G C G T A C G A T A T C G C G A T | 1e-41 | -96.619429 | 4.47% | 1.83% | motif file (matrix) |
| 3 | 0.796 | A T G C G C A T A C G T G C A T A C T G C G T A G C A T A T C G A C G T A C T G | 1e-40 | -94.168974 | 2.81% | 0.86% | motif file (matrix) |
| 4 | 0.782 | A C G T A C G T A C G T A C T G C G T A A G C T A T C G A C G T | 1e-33 | -76.306278 | 3.17% | 1.22% | motif file (matrix) |
